# Exploring the effects of a combined exercise programme on pain and fatigue outcomes in people with systemic sclerosis: study protocol for a large European multi-centre randomised controlled trial

**DOI:** 10.1186/s13063-022-06853-1

**Published:** 2022-11-28

**Authors:** Alexandros Mitropoulos, Carina Boström, Malin Mattsson, Evangelia Kouidi, Theodoros Dimitroulas, Sophie I. E. Liem, Theodora P. M. Vliet Vlieland, Jeska K. de Vries-Bouwstra, Søren Jacobsen, Giovanna Cuomo, Mohammed Akil, Markos Klonizakis

**Affiliations:** 1 Lifestyle, Exercise and Nutrition Improvement (LENI) Research Group, Department of Nursing and Midwifery, Sheffield Hallam University, Sheffield, UK; 2 Department of Neurobiology, Care Sciences and Society, Karolinska Institutet, Stockholm, Sweden; 3 Laboratory of Sports Medicine, Department of Physical Education and Sports Sciences, Aristotle University of Thessaloniki, Thessaloniki, Greece; 4 4th Department of Internal Medicine, School of Medicine, Hipokration Hospital, Aristotle University of Thessaloniki, Thessaloniki, Greece; 5 Department of Rheumatology, Leiden University Medical Center, Leiden, The Netherlands; 6 Copenhagen Research Centre for Autoimmune Connective Tissue Diseases, Copenhagen University, RigshospitaletCopenhagen, Denmark; 7 Department of Precision Medicine, University of Campania L. Vanvitelli, Naples, Italy; 8 Department of Rheumatology, Royal Hallamshire Hospital, Sheffield, UK

**Keywords:** Systemic sclerosis, Pain, Fatigue, Exercise, High-intensity interval training, Microvascular function, Quality of life, Aerobic, Muscular function

## Abstract

**Background:**

Pain, related to Raynaud’s phenomenon or digital ulceration, has been identified as very prevalent and debilitating symptoms of systemic sclerosis (SSc), both significantly affecting patients’ quality of life (QoL). Pharmacological therapeutic strategies were found not to be sufficiently effective in the management of SSc-induced pain and fatigue, and evidence for exercise is scarce. As yet, the effects of a long-term, tailored exercise programme on pain and fatigue in patients with SSc have not been explored. In addition to pain and fatigue, this study aims to evaluate the effects of exercise on QoL, physical fitness, functional capacity, and vascular structure in people with SSc (PwSSc).

**Methods:**

This will be a multicentre (*n* = 6) randomised controlled clinical trial to assess the effect of a previously established, supervised 12-week combined exercise programme on pain and fatigue as compared to no exercise in PwSSc. The study will recruit 180 patients with SSc that will be allocated randomly to two groups. Group A will perform the exercise programme parallel to standard usual care and group B will receive usual care alone. Patients in the exercise group will undertake two, 45-min sessions each week consisting of 30-min high-intensity interval training (HIIT) (30-s 100% peak power output/30-s passive recovery) on an arm crank ergometer and 15 min of upper body circuit resistance training. Patients will be assessed before as well as at 3 and 6 months following randomisation. Primary outcomes of the study will be pain and fatigue assessed via questionnaires. Secondary outcomes include quality of life, structure of digital microvasculature, body composition, physical fitness, and functional capacity.

**Discussion:**

Data from this multi-centre research clinical trial will primarily be used to establish the effectiveness of a combined exercise protocol to improve pain and fatigue in SSc. In parallel, this study will be the first to explore the effects of long-term exercise on potential microvascular alterations assessed via NVC. Overall, this study will provide sufficient data to inform current clinical practice guidelines and may lead to an improvement of QoL for patients with SSc.

**Trial registration:**

ClinicalTrials.gov NCT05234671. Registered on 14 January 2022

**Supplementary Information:**

The online version contains supplementary material available at 10.1186/s13063-022-06853-1.

## Background

Systemic sclerosis (SSc) is a rare autoimmune disease of unknown aetiology characterised by skin fibrosis and internal organ and vascular involvement. SSc is more common to women compared to men with a ratio of almost 5:1 to be consistently reported in the majority of studies [1]. Geographically, the incidence and prevalence of SSc varies across Europe. More specifically, a North-South gradient in Europe has been previously proposed [2] with Northern European countries such as the UK, Finland, and Iceland presenting lower rates compared to Southern European countries such as France and Greece.

Pain and fatigue [3] are two of the most common and important patient-reported outcomes in rheumatology [3] and SSc [4]. Several organ systems and factors are responsible for the origination of both pain (e.g. vascular, dermal, musculoskeletal, gastrointestinal, genitourinary) and fatigue (e.g. cardiac, respiratory, muscular, systemic inflammation, psychological, neurological, malnutrition, sleep-related, and medication-related) [4]. Both symptoms (i.e. pain and fatigue) are strongly linked to poor quality of life (QoL) in people with SSc [4].

Pharmacological therapeutic strategies [5] have been adopted in the management of pain and fatigue in SSc without being sufficiently effective [6–8]. The evidence of the effectiveness of non-pharmacological interventions (e.g. the effects of exercise on SSc pain and fatigue) is limited [9, 10], but promising [11–15]. Engagement in regular exercise is associated with lower pain intensity and interference in people with SSc [16] (PwSSc). Higher daily physical activity levels are associated with improved skin microvascular function in type 2 diabetes [17] (a clinical condition with increased vasculopathy similar to that observed in SSc). This might explain partly why RP’s severity (i.e. pain) and presence of ulcers are observed less in toes (as lower limbs are the most commonly used body part for daily activities) when compared to fingers [18].

Physically active PwSSc have lower levels of fatigue compared to inactive individuals [16]. Moreover, evidence (e.g. one-to-one semi-structured interviews) from our recently completed feasibility study [12] suggests that PwSSc feel more energetic and stronger following a combined (e.g. aerobic and resistance training) exercise programme, also improving their fitness and social life [12]. Thus, a feasible, safe, long-term exercise programme for PwSSc that will improve pain and fatigue is warranted. Therefore, and being supported by published evidence [12–14], the aim of the current European randomised controlled clinical trial (RCT) is to assess the effect of such an exercise programme primarily on pain and fatigue, as well as on QoL, physical fitness, upper body functional capacity, and digital vascular structure in PwSSc.

## Methods

### Study design

**Fig. 1:**
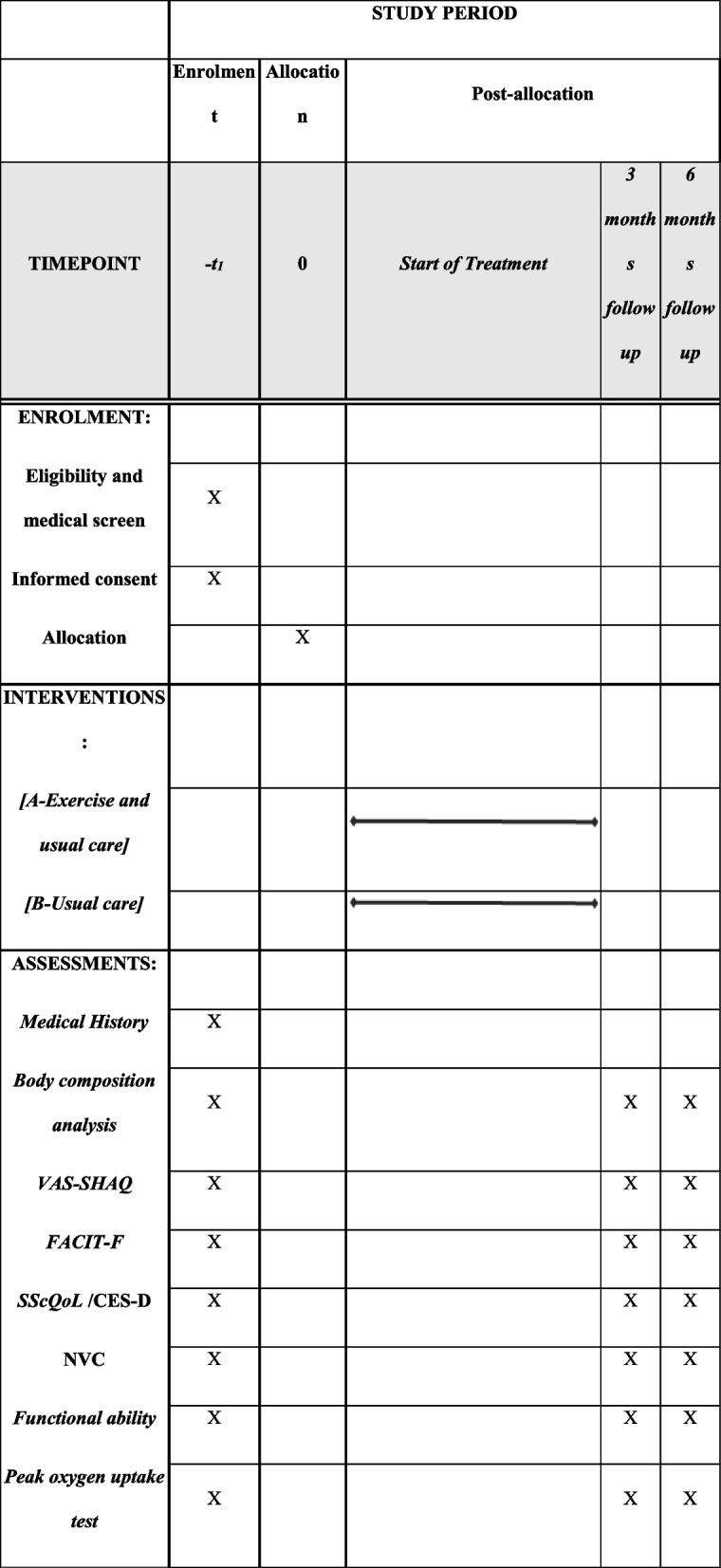
Study design. VAS-SHAQ, Visual Analogue Scale-Scleroderma Health Assessment Questionnaire; FACIT-F, functional assessment of chronic illness therapy—fatigue; SScQoL, Systemic Sclerosis Quality of Life Questionnaire; CES-D, Centre for Epidemiologic Studies Depression Scale; NVC, nailfold videocapillaroscopy

The CESPF (The effects of a combined supervised exercise programme on pain and fatigue in SSc) study is a multi-centre (*n*=6) randomised (1:1 ratio), parallel group, superiority, controlled clinical trial. One hundred and eighty SSc will be recruited across six research institutions (Sheffield Hallam University, Sheffield, UK; Karolinska Institutet, Stockholm, Sweden; Rigshospitalet Hospital, Copenhagen, Denmark; Leiden University Medical Center, Leiden, Netherlands; Aristotle University, Thessaloniki, Greece; University of Campania L. Vanvitelli, Naples, Italy) and will be randomly assigned via stratified block randomisation remotely by an independent statistician to either group A (combined exercise) or group B (control group). Both groups will continue receiving their usual medical as well as non-pharmacological treatment (if applicable) throughout the study. Patients will be followed for 6 months following randomisation (Fig. 1). Assessors will be blinded regarding the allocated treatments. The study’s protocol and registration have been published in ClinicalTrials.gov (NCT number: NCT05234671) January 02, 2022. The trial is sponsored by the Foundation for Research in Rheumatology, and medical (where applicable) or non-medical ethical approval has been granted by each research centre locally. The reporting of this study protocol is done according to the Standard Protocol Items Recommendations for Interventional trials (SPIRIT).

### Recruitment of participants

The RCT will recruit 180 (exercise group *n* = 90 and control group *n* = 90) PwSSc from the above-mentioned six European study/recruitment centres via local rheumatological clinics. PwSSc who meet the eligibility criteria (see below) and express an interest will receive a detailed patient’s information sheet from the treating health care professional (e.g. rheumatologist, physiotherapist, nurse). Following a certain time period dependant on each country’s ethical regulations, a study investigator will contact them either via letter or telephone to address any queries regarding the study and arrange an initial appointment. Clinical care will not be affected for those not wishing to take part or withdraw from the study. Depending on the conditions of local ethical approvals, at the first visit, participants will be familiarised with the study’s procedures, provide informed consent, and complete baseline assessments. Informed consent will be performed by the local designated principal investigator.

### Eligibility criteria

Patients eligible for the trial must comply with all the following inclusion and exclusion criteria:

#### Inclusion criteria

- A diagnosis of SSc according to the 2013 American College of Rheumatology/European League Against Rheumatism criteria and experiencing RP
- Age over 18 years old
- Ability to perform the prescribed exercise regime

#### Exclusion criteria

- Active disease-related exacerbations (e.g. active digital ulcers)
- Change of medical vascular function-related treatment (i.e. vasodilators) within the last 3 months
- Advanced pulmonary involvement (e.g. pulmonary arterial hypertension)
- New York Heart Association class 3 or 4
- Inability to exercise
- Current pregnancy

### Group randomisation

Following VISIT 1, participants will be randomised remotely (to ensure allocation and concealment) into groups A (combined exercise) and B (control group), using a computer programme (nQuery Advisor 6.0, Statistical Solutions, Ireland) to generate stratified block-randomisation (by research centre, SSc-type, disease duration and severity), by an independent statistician. Each participant will be allocated a unique trial number across all sites (e.g. SSc001) that will remain with them throughout the study.

### Exercise intervention

The exercise intervention will require patients to be randomly allocated into two groups. Groups A and B will serve as the exercise and control groups, respectively. The comparison between those who will perform an individualised combined (aerobic and resistance training) exercise programme with supervised sessions and those who will not perform the exercise programme is essential to examine whether exercise is beneficial and effective for PwSSc.

Following randomisation, group A will perform a 12-week combined exercise programme (aerobic and resistance training) twice per week. The exercise programme will be performed adjunctive to usual care. Each session will be supervised and will consist of 3- to 5-min warm up performing light to moderate intensity arm cranking (55–65% of peak power output; PPO), 30-min high-intensity interval training (HIIT; 30 s at 100% of PPO and 30-s passive recovery) and 3 to 5 min of cool down period (40–50% of PPO) on an arm crank ergometer. The PPO will be individualised tested when performing the peak exercise test on an arm crank ergometer at baseline assessments (see assessments section). The HIIT protocol will be combined with resistance training (RT) lasting for a total of approximately 15 min. RT will be consisted of an upper body circuit training (five exercises): (1) shoulder lateral raise in a sitting position with dumbbells, (2) chest press on a bench in a 30° supine position with dumbbells or chest press machine, (3) biceps curl in a sitting position with dumbbells, (4) triceps extension in a sitting position with dumbbell or standing position in a pulley machine with straight grip, and (5) hand squeeze of a handgrip dynamometer, for three circuits. The intensity for all the exercises except the handgrip dynamometer will be at 75–80% of one repetition maximum (1-RM; that will be determined indirectly via validated 1-RM prediction formulas [19]) performing 10 repetitions of each exercise interspersed by 20–30 s to allow for safe movement between exercises. For the handgrip dynamometer exercise, the intensity will be maximum for 10 repetitions. The recovery period between circuits will last 2–3 min. Following each exercise session, patients will undertake a 5-min cool-down period, involving arm cranking (e.g. corresponding to “light intensity” based on rating of perceived exertion; RPE [20]) and some light stretching with focus on pectoral and deltoid muscles (3–4 repetitions to each arm for 20–30 seconds). Heart rate (HR; using a heart rate monitor chest strap) and RPE will be monitored at regular intervals (both during the exercising bouts as well as during the passive recovery approximately every 5 min) throughout the exercise session. Blood pressure (BP) will be monitored pre- and post- each session. A qualified health care professional (e.g. clinical exercise physiologist/physical therapist with experience in SSc) will supervise the exercise session.

Any potential adverse events (e.g. pain) during the exercise sessions will be assessed for relation with the intervention and will be recorded by the health care professional.

### Control group

Group B will not perform any exercise intervention and will only receive the usual care. To assure the provision of equal opportunities for both groups, following VISIT 3 (e.g. meaning the completion of the research protocol for the participants), group B will be offered the same exercise intervention as in group A.

### Withdrawals

Patients will be considered as withdrawn from the research trial if they request to leave the trial if they are lost to follow-up or if they die before completing the 6-month follow-up. Discontinuation of the allocated intervention will be applied in case of adverse events that may compromise patient’s health status or reduce health related QoL.

#### Six pillars of adherence

The 6 pillars of adherence will be used to support the feasibility, recruitment, and participation enjoyment levels of PwSSc. This approach has been successfully applied in our research group’s previous clinical trials with PwSSc [12, 21], developing thus a standard quality service.

“Social support”: Participants are encouraged to speak to family members if they wish to, and if the implemented health protocols allow it, they can bring a friend or family member together with them at assessments. Research has showed that those who receive support to continue from a member of their inner circle are usually the ones who complete treatments successfully.

“Education”: Participants are educated for the benefits of exercise and healthy diet. The focus lies primarily on the overall health and wellbeing benefits, explaining also in lay terms that recent work suggests that there is a clinical outcome potential (fewer digital ulcers and hospitalisations) [13].

“Reachability”: Reachability is the ascertaining to participants of easy and quick contact (i.e. phone, email, social media) to provide them valuable support. This allows study’s participants to reach the research team outside the exercise sessions with questions, queries, and advice requests.

“Small groups intervention implementation”: Wherever that is possible (i.e. depending on available training equipment) small groups are formed, where the same people on each session, exercise together, supporting each other, trying to create a social element supporting thus adherence to exercise training.

“Reminders”: Apart from the initial provision of a detailed programme, participants receive reminders of their upcoming sessions via e-mail and/or text, at least one day before their exercise sessions.

“Simplicity”: The fitness goals are kept simple, realistic, and individualised based on individualised exercise testing. Participants are receiving appropriate education of the importance to set simple attainable goals.

### Assessments

Assessments will be performed at baseline (i.e. VISIT 1), at 3 months (i.e. VISIT 2) and at 6 months (i.e. VISIT 3). Assessments will consist of questionnaires and physical tests. All physical tests are administered by trained assessors, who are blinded to the patients’ randomisation status. To ensure high-quality standards of the proposed assessments, the study’s research manager (AM) will provide across centres a common standard operation procedure (SOP) digital manual for all baseline measurements (including follow-up visits), as well as day-to-day quality monitoring procedures.

### Outcomes

#### Primary outcomes

##### Pain (overall and digital)

Overall pain will be assessed using the VAS-SHAQ (Additional file 1) [22]. VAS-SHAQ is consisted of a 15-cm which is converted to a continuous scale from 0 to 3 (1cm = 0.2 points on the VAS). Digital pain will be assessed using a unidimensional measure of pain intensity, which has been widely used in diverse adult populations, including those with rheumatic diseases [23]. The pain VAS will be comprised of a horizontal line,100 mm in length, anchored by 2 verbal descriptors, one for each symptom extreme: “no pain” (score of 0) and “worst imaginable pain” (score of 100) [23]. For both VAS-SHAQ and digital VAS scales, participants will be requested to self-complete the pain by placing a line perpendicular to the VAS line at the point that represents their overall and digital pain intensity, respectively.

##### Fatigue

Fatigue will be assessed using the functional assessment of chronic illness therapy—fatigue (FACIT-F [24]; Additional file 2) which has also been utilised in studies assessing fatigue in people with SSc [9, 25]. Participants will be requested to self-complete this 40-item questionnaire which will assess fatigue and its impact on daily activities and function. FACIT-F is formatted in a Likert-type scale (0 = not at all; 1 = a little bit; 2 = somewhat; 3 = quite a bit; and 4 = very much). All items contribute to the sum score with equal weight. The scale range is 0 to 52, with 0 being the worst possible score and 52 being the best possible score indicating no fatigue.

#### Secondary outcomes

##### Quality of life

SSc-related QoL will be assessed using the SScQoL questionnaire [26] (Additional file 3), and the CES-D (Additional file 4) [27] will be used to assess the depressive symptoms. The SScQoL is a questionnaire comprising 29 questions exploring the impact of SSc on health and well-being, covering four themes identified by PwSSc: emotion, physical adaptation, impact on/with others, and impact on self.

The Centre for Epidemiologic Studies Depression Scale (CES-D) has shown to be a reliable and valid measure of depressive symptoms in patients with SSc [28]. The CES-D is a 20-item measure, with the frequency of each depressive symptom rated on a 4-point Likert scale ranging from 0 to 3 (“rarely or none of the time” to “most or all of the time”).

##### Digital cutaneous microvascular structure

NVC will be performed using an optical probe (200× contact lenses) connected to image analysis software. Images will be observed on a high-resolution colour monitor. Patients will be instructed to wash their hands gently with an antibacterial soap, and following 15 min of rest (i.e. seated), NVC will be performed at a standardised room temperature of 20–25°C. The nailfolds of six digits (e.g. index, middle, and ring in both hands) will be examined in each patient, and a drop of vegetable oil will be placed on the nailfold bed to improve the image resolution. Digits that will be affected by severe local trauma will not be analysed. The following parameters will be recorded and analysed: scleroderma patterns (e.g. early, active, late), presence of enlarged and giant capillaries, haemorrhages, loss of capillaries, disorganisation of the vascular array, and ramified/bushy capillaries [29]. A qualitative and semi-quantitative scoring of SSc patterns by NVC will be performed as described and validated previously [30, 31].

Prior to NVC assessment, patients will be instructed to avoid from caffeine and smoking for at least 4 h and to not remove the fingernail cuticles for at least 2–3 weeks to avoid microtraumas that could affect the examination.

### Peak oxygen uptake test

Throughout the test, gas exchange will be collected and analysed by an online breath-by-breath analysis system. Heart rate (HR) and the electrocardiogram (ECG) will be continuously monitored. RPE (6–20 points [20]) will be recorded during the last 10 s of every minute during the exercise test until volitional exhaustion. Blood pressure will be assessed prior and 5 min following the termination of the exercise test to assure resting values have been reached. Peak power output (PPO) measured in watts and test duration (e.g. minutes) will be recorded. Peak oxygen uptake (V̇O_2peak_) will be defined as the average oxygen during the last 30s. The incremental exercise protocol will be calculated based on patient’s physical activity levels. Physical activity is assessed via certain questions (e.g. regular physical activities or exercise training, duration of each session, intensity, and mode of exercise) concerning the last 4 weeks. The crank rate will be maintained above 50 rev min^−1^ (patient’s will be allowed to select and maintain their own comfortable crank rate), and power requirements will be increased as a linear ramp at a rate of 10 W/min and 6 W/min for males and females, respectively [32, 33]. The test will be commenced with 2 min collecting resting values and 3 min of warm-up (unloaded cranking). RPE ≥18 and/or inability to maintain a crank rate above 50 rev min^-1^ will be resulted in the termination of the test. After the exercise termination, an unloaded or very low loaded (e.g. <5watts) bout of 2–3 min exercise at a crank rate below 40 rev min^−1^ will follow to allow for an active recovery period.

### Handgrip test

Southampton protocol for adult grip strength measurement will be utilised [34], and participants will be familiarised with procedures prior to testing. The participant will be seated in a standard chair with legs, back support, and fixed arms. The same chair will be utilised for every measurement. The forearms will be placed on the arms of the chair with the wrist just over the end of the arm of the chair (i.e. wrist in a neutral position, thumb facing upwards). Jamar handgrip dynamometer will be positioned as such so as the thumb is round one side of the handle, and the four fingers are around the other side. The assessor will rest the base of the dynamometer on the palm of their hand as the subject holds the dynamometer (to negate the effect of gravity on peak strength). The participant will be instructed to squeeze as long and as tightly as possible or until the needle stops rising. Once the needle stops rising, the participant will be instructed to stop squeezing. Strength will be reported in kilogrammes. The measurement will be repeated in the left hand. Three attempts will be performed in total for each side and the best of the three grip strength measurements will be recorded. Moreover, hand dominance (e.g. right, or left) or ambidextrous (i.e. people who can genuinely write with both hands) will also be recorded.

### Arm curl test

The aim of this test is to do as many arm curls as possible in 30 s. This test will be conducted on the dominant arm side. The participant will be seated on the chair, holding the weight in the hand with palm facing towards the body and the arm in a vertically down position beside the chair. The upper arm will be supported against the body so that only the forearm will be moving. The arm will be fully bent and then fully straightened at the elbow. The arm curl test will require the subject to repeatedly lift a 2-kg weight (for women) or a 4-kg weight (for men) for 30 s [35]. The number of lifts will be recorded.

### Clinical management

All patients in both groups will continue receiving their standard medical treatment such as calcium channel blockers (e.g. nifedipine, sildenafil) and will be reviewed in the clinic as often as is deemed clinically necessary.

Moreover, in case of adverse events or worsening of symptoms due to exercise, patients will be withdrawn from the study for health and safety reasons. Patients can also withdraw on own request.

### Data management

The study will adhere to the Data Protection Act (1998) and to the General Data Protection Regulation (GDPR). Data from this study will be pseudonymised and stored in password-protected computer systems or institutional secure drivers accessible only by the members of each local research team in order to guarantee confidentiality to patients. Paper forms will be stored in locked filing cabinets. Data will be kept at Sheffield Hallam University for a 7-year period following the study completion to allow the study team to answer all the research questions.

All data documents will be kept in secured and continuously monitored buildings. All study materials will remain in this location for data entry and storage. Participant names will not be used to identify any data. Instead, participants will be assigned a study identification number (e.g. SSc001) in order to pseudonymise their information and thus all the related collected data. Publications will not contain identifiable personal data. Furthermore, only local research principal investigators will have access to personal patient data during the project, while data will be analysed locally. Following data collection and local data analysis, all research centres will securely (e.g. via drivers) transfer the data to Sheffield Hallam University to perform the statistical analysis. All centres will have digital secure access to the final aggregated large database.

### Data monitoring

The data monitoring committee (DMC) will be comprised of the research manager and chief investigator of the leading research centre (i.e. Sheffield Hallam University) due to the low risk of the intervention. The conduct of the study will be overlooked on a daily basis by the chief investigator Dr Alexandros Mitropoulos at Sheffield Hallam University. Research meetings will be held between all members of the research team once monthly to discuss further progress and set targets. The DMC is independent from the sponsor and has no competing interests.

Moreover, a designated member of the research team will conduct audit trials to the study’s centres to explore whether the study is running according to the SOP at regular intervals. This audit will be run independently of the sponsor.

### Safety monitoring

We will record all serious adverse events (as defined below), as well as all non-serious adverse events that are deemed to be related to participation in the research, during the period between the provision of informed consent through to 6 months after randomisation.

Serious adverse events are defined as any untoward medical occurrence that either results in death, is life-threatening (i.e. the patient is at risk of death at the time of the event occurring), requires unplanned admissions to emergency hospitalisations or prolonged hospitalisation (deemed to be where a patient’s stay is longer than expected, e.g. patient is operated on as a day case but remains in hospital overnight) or results in persistent or significant disability or incapacitation.

A non-serious event in the context of this trial will be any untoward medical occurrence to the participant that is related to the patients’ involvement in the study but does not fulfil any of the serious adverse event criteria.

### Statistical analysis

Data analysis will be performed using SPSS software (IBM SPSS, New York, USA) and will be presented as mean ± SD. Normal distribution of the data and homogeneity of variances will be tested using the Shapiro-Wilk and Levene’s test, respectively. The comparison between the two groups for pain and fatigue (primary outcomes) as well as NVC, functional capacity tests, and V̇O_2peak_ test (secondary outcomes) will be performed through independent *t*-tests, Wilcoxon, chi-squared, Mann-Whitney *U*, and Kruskal-Wallis tests. Mixed model ANCOVA will also be performed to test the differences both within and between subject effects across time [three measurements: baseline, 12 weeks (primary time-point), and 24 weeks post-baseline]. Effect sizes (Cohen’s *d*) will be calculated wherever the results will be statistically significant with 0.2, 0.5, and 0.8 representing small, medium, and large effects respectively [36]. Statistical significance will be set at *p* ≤ 0.05. Data analysis will commence at the end of the 2nd data collection post-baseline (i.e. 12 weeks).

#### Sample size

The primary outcome was the RP pain. For our calculations, we used commercial software (G*Power 3.1.7, HHU of Düsseldorf) by using data from two studies that examined the exercise training effects on SSc-QoL [35, 37]. Based on those calculations, we will need no more than 90 patients in each group (180 in total) to detect a difference in RP’s pain at 3 months (significance level = 0.05; power =80%) accounting also for an estimated 15% dropout and 5% site effect.

## Discussion

Pain and fatigue are two of the most common symptoms in PwSSc. Both pain and fatigue in SSc are associated with more severe disease, depression, and poor QoL and adversely affect the functional ability, social, and emotional wellbeing. As yet, we lack strong evidence concerning the effects of exercise on pain and fatigue outcomes. Regular exercise has demonstrated promising results; however, a large research clinical trial is warranted to establish the effects of exercise. For that reason, the findings of the current study will inform the future clinical disease management and will play a critical role in clinical decision-making in people with SSc.

Two of the most common causes of pain in SSc are the joint and RP pain, and less commonly reported the painful digital ulcers [37]. RP pain is strongly linked with the endothelial dysfunction and inflammatory cascade leading to an impaired blood flow and tissue perfusion [38]. In PwSSc, both aerobic alone as well as a combined (e.g. aerobic and resistance training) exercise protocol have demonstrated improvements in the digital endothelial function which also depicted as a prevention of digital ulcers and a decrease in RP pain and severity (i.e. intensity and duration) [12–14], in the exercise group (i.e. 12-week exercise programme; twice per week) compared to the control group. With respect to the joint pain, evidence suggests that aerobic and strength training are able to reduce knee pain associated with osteoarthritis [39]. Moreover, it has been demonstrated that muscle weakness is associated with increased pain levels and poorer physical function in osteoarthritis [40, 41]. Future research studies shall examine the associative physiological outcome (e.g. peak oxygen uptake or muscle mass/strength assessed via body composition analysis and muscle strength tests) to the pain relief following exercise.

Fatigue in SSc is associated with poor QoL (e.g. work disability, depression, sleeping disorders, pain, physical function, social activity) [9]. Pharmacological interventions have been proved ineffective to improve fatigue in SSc; however, physical activity (mainly walking) [16] and a combined exercise (i.e. aerobic and resistance training, three times per week for 8 weeks) [42] have demonstrated that they are able to reduce fatigue levels in SSc. In addition, following a 12-week combined supervised individualised exercise programme (twice per week), PwSSc reported that they felt more active and awake performing their daily tasks with less effort [12]. The pathophysiology of fatigue is not well understood; however, several causative factors have been identified, among which, cytokine imbalance, sleep disturbances, and deconditioning [43]. Exercise has shown that it is able to improve the anti-inflammatory immune response [44]. Moreover, cardiorespiratory fitness (i.e. peak oxygen uptake) is strongly linked to sleep quality [45] and in improving physical conditioning. Evidently cardiorespiratory deconditioning (i.e. low peak oxygen uptake) may play a role in the development and persistence of cancer-related fatigue following treatment [46].

The pathogenesis of SSc is complex and includes vascular alterations (i.e. vasculopathy), immune system dysregulation, and aberrant tissue fibrosis [47, 48]. Morphological and functional analysis of the microcirculation are objective outcome measures that are recommended for use in the presence of clinical signs of altered peripheral blood flow (such as Raynaud phenomenon), which can occur in SSc. NVC is the best-studied and most commonly used method for distinguishing and quantifying microvascular morphological alterations in SSc. This study will aim to assess the effects of long-term (e.g. 3-month) combined exercise programme on the microvascular morphological alterations using NVC.

Overall, the study is expected to provide valuable evidence on the effects of exercise on pain and fatigue and whether these two outcomes could be managed with a feasible (i.e. for people with SSc) combined exercise protocol. Our study will also aim to identify any potential correlations between several physiological outcomes (e.g. peak oxygen uptake, vascular structure, strength tests, body composition analysis) and potential improvements in pain and fatigue in PwSSc. 

### Practical issues

The current study is expected to overcome some practical issues that might appear as a result of the multi-centre collaboration. Namely, local ethical approvals were the first practical issue that has now been overcome and recruitment has been initiated in all European centres. Completing recruitment on time is the next challenge of this multi-centre trial; nevertheless, all centres and investigators have got the experience and the appropriate number of people with SSc to contribute effectively to the recruitment target (i.e. approximately 30 participants per centre). Another practical issue is the standardisation of the baseline and follow-up assessments across all centres. The standardisation has been secured by creating a standard operating procedures manual which is strictly followed by all research centres and investigators.

## Trial status

Protocol version 2.0, 6 September 2022. Recruitment started in December 2021 and is expected to be completed by end of January 2023.

## Supplementary Information

**Additional file 1.** VAS-SHAQ Questionnaire

**Additional file 2. **FACIT-F Questionnaire 

**Additional file 3. **SscQoL Questionnaire 

**Additional file 4. **CES-D Questionnaire  

## Data Availability

Access to the final trial database will be granted to all Principal Investigators from the participating research institutions.

## Abbreviations

BP: Blood pressure
CES-D: Centre for epidemiologic studies-depression scale
ECG: Electrocardiogram
FACIT-F: Functional assessment of chronic illness therapy—fatigue
GDPR: General Data Protection Regulation
HIIT: High-intensity interval training
HR: Heart rate
NVC: Nailfold videocapillaroscopy
PPO: Peak power output
PwSSc: People with systemic sclerosis
QoL: Quality of life
RCT: Research clinical trial
RM: Repetition maximum
RP: Raynaud’s phenomenon
RPE: Ratings of perceived exertion
RT: Resistance training
SSc: Systemic sclerosis
SOP: Standard operation procedures
SScQoL: Systemic Sclerosis Quality of Life Questionnaire
VAS-SHAQ: Visual Analogue Scale-Scleroderma Health Assessment Questionnaire
