# Supplementary material for: Exploring the effects of a combined exercise programme on pain and fatigue outcomes in people with systemic sclerosis: study protocol for a large European multi-centre randomised controlled trial
Source: Trials. 2022 Nov 28;23:962. doi: 10.1186/s13063-022-06853-1 (PMC9706982; doi:10.1186/s13063-022-06853-1)
Supplement: Supplementary file 1 — Additional file 1. VAS-SHAQ Questionnaire [file 13063_2022_6853_MOESM1_ESM.pdf]

HEALTH ASSESSMENT QUESTIONNAIRE

In this section we are interested in learning how your illness affects your ability to function in daily life. Please feel free to add comments.

Please check the one response that best describes your usual abilities
IN THE PAST SEVEN DAYS:

	Without **ANY** difficulty	With **SOME** difficulty	With **MUCH** difficulty	**UNABLE** to do
**DRESSING & GROOMING**				
Are you able to:				
- Dress yourself, including tying shoelaces and doing buttons	____	_______	______	_____
- Shampoo your hair?	_____	_____	_____	_____
**ARISING**				
Are you able to:				
- Stand up from an armless straight chair?	_____	_____	_____	_____
- Get in and out of bed?	_____	_____	_____	_____
**EATING**				
Are you able to:				
- Cut your meat?	_____	_____	_____	_____
- Lift a full glass to your mouth?	_____	_____	_____	_____
- Open a new milk carton?	_____	_____	_____	_____
**WALKING**				
Are you able to:				
- Walk outdoors on flat ground?	_____	_____	_____	_____
- Climb up five stairs?	_____	_____	_____	_____

Please check any **AIDS or DEVICES** that you usually use for any of these activities:

____Cane	___Devices for dressing (button hook, zipper pull, long-handled shoe horn, etc.)
____Walker	___Built up or special utensils
____Crutches	___Special or built-up chair
____Wheelchair	___Other (specify: _________________)

Please check any categories for which you usually need **ASSISTANCE FROM ANOTHER PERSON:**

_______ Dressing and grooming	________ Eating
_______ Arising	________ Walking

HEALTH ASSESSMENT QUESTIONNAIRE

In this section we are interested in learning how your illness affects your ability to function in daily life. Please feel free to add comments.

Please check the one response that best describes your usual abilities
IN THE PAST SEVEN DAYS:

	Without **ANY** difficulty	With **SOME** difficulty	With **MUCH** difficulty	**UNABLE** to do
**HYGIENE**				
Are you able to:				
- Wash and dry your entire body?	_____	_____	_____	_____
- Take a tub bath?	_____	_____	_____	_____
- Get on and off the toilet?	_____	_____	_____	_____
**REACH**				
Are you able to:				
- Reach and get down a 2.5kg object (such as a bag of sugar) from just over your head?	_____	_____	_____	_____
- Bend down and pick up clothing off the floor?	_____	_____	_____	_____
**GRIP**				
Are you able to:				
- Open car doors?	_______	_______	___	_______
- Open jars that have been previously opened?	_______	_______	___	_______
- Turn faucets on and off?	_______	_______	___	_______
**ACTIVITIES**				
Are you able to:				
- Run errands and shop?	_______	_______	___	_______
- Get in and out of a car?	_______	_______	___	_______
- Do chores such as vacuuming or yardwork?	_______	_______	___	_______

Please check any **AIDS or DEVICES** that you usually use for any of these activities:

___Raised Toilet Seats ___Bathtub Bar ___Bathtub Seat

___Long-Handled Appliances for Reach ___Jar Opener (for jars previously opened)

___Long-Handled Appliances in Bathroom Other (specify: ______________________)

Please check any categories for which you usually need **HELP FROM ANOTHER PERSON:**

____Hygiene ____Gripping and Opening Things

____Reach ____Errands and Chores

We are also interested in learning whether or not you are affected by pain because of your illness. How much pain have you had because of your illness **IN THE PAST WEEK?**

PLACE A MARK ON THE LINE TO INDICATE THE SEVERITY OF THE PAIN.

NO PAIN VERY SEVERE PAIN

0 100

IN THE PAST WEEK, how much have your intestinal problems interfered with your daily activities?

PLACE A MARK ON THE LINE TO INDICATE THE LIMITATION OF ACTIVITY.

DO NOT LIMIT ACTIVITIES VERY SEVERE LIMITATION

0 100

IN THE PAST WEEK, how much have your breathing problems interfered with your daily activities?

PLACE A MARK ON THE LINE TO INDICATE THE LIMITATION OF ACTIVITY.

BREATHING PROBLEMS
DO NOT LIMIT ACTIVITIES

VERY SEVERE
LIMITATION

0 100

IN THE PAST WEEK, how much has Raynaud's interfered with your daily activities?

PLACE A MARK ON THE LINE TO INDICATE THE LIMITATION OF ACTIVITY.

RAYNAYD'S DOES
NOT LIMIT ACTIVITIES

VERY SEVERE
LIMITATION

0 100

IN THE PAST WEEK, how much have your finger ulcers interfered with your daily activities?

PLACE A MARK ON THE LINE TO INDICATE THE LIMITATION OF ACTIVITY.

FINGER ULCERS
DO NOT LIMIT ACTIVITIES

VERY SEVERE
LIMITATION

0 100

Overall, considering how much pain, discomfort, limitations in your daily life and other changes in your body and life,

how severe would you rate your disease today?

PLACE A MARK ON THE LINE TO INDICATE THE LIMITATION OF ACTIVITY.

NO DISEASE

VERY SEVERE LIMITATION

0

100

Steen VD, Medgers TA. The Value of the Health Assessment Questionnaire and special patient-generated scales to demonstrate change in systemic sclerosis patients over time. Arthritis Rheum 1997 Nov ; 40 (11):1984–1991.
