# Supplementary material for: Exploring the effects of a combined exercise programme on pain and fatigue outcomes in people with systemic sclerosis: study protocol for a large European multi-centre randomised controlled trial
Source: Trials. 2022 Nov 28;23:962. doi: 10.1186/s13063-022-06853-1 (PMC9706982; doi:10.1186/s13063-022-06853-1)
Supplement: Supplementary file 2 — Additional file 2. FACIT-F Questionnaire [file 13063_2022_6853_MOESM2_ESM.pdf]

FACIT-F (Version 4)

Below is a list of statements that other people with your illness have said are important. **Please circle or mark one number per line to indicate your response as it applies to the <u>past 7 days</u>.**

	PHYSICAL WELL-BEING	Not at all	A little bit	Some-what	Quite a bit	Very much
GP1	I have a lack of energy	0	1	2	3	4
GP2	I have nausea	0	1	2	3	4
GP3	Because of my physical condition, I have trouble meeting the needs of my family	0	1	2	3	4
GP4	I have pain	0	1	2	3	4
GP5	I am bothered by side effects of treatment	0	1	2	3	4
GP6	I feel ill	0	1	2	3	4
GP7	I am forced to spend time in bed	0	1	2	3	4

	SOCIAL/FAMILY WELL-BEING	Not at all	A little bit	Some-what	Quite a bit	Very much
GS1	I feel close to my friends	0	1	2	3	4
GS2	I get emotional support from my family	0	1	2	3	4
GS3	I get support from my friends	0	1	2	3	4
GS4	My family has accepted my illness	0	1	2	3	4
GS5	I am satisfied with family communication about my illness	0	1	2	3	4
GS6	I feel close to my partner (or the person who is my main support)	0	1	2	3	4
Q1	*Regardless of your current level of sexual activity, please answer the following question. If you prefer not to answer it, please mark this box* ☐ *and go to the next section.*					
GS7	I am satisfied with my sex life	0	1	2	3	4

Copyright 1987, 1997

FACIT-F (Version 4)

Please circle or mark one number per line to indicate your response as it applies to the <u>past 7 days</u>.

	**<u>EMOTIONAL WELL-BEING</u>**	**Not at all**	**A little bit**	**Some-what**	**Quite a bit**	**Very much**
GE1	I feel sad	0	1	2	3	4
GE2	I am satisfied with how I am coping with my illness	0	1	2	3	4
GE3	I am losing hope in the fight against my illness	0	1	2	3	4
GE4	I feel nervous	0	1	2	3	4
GE5	I worry about dying	0	1	2	3	4
GE6	I worry that my condition will get worse	0	1	2	3	4

	**<u>FUNCTIONAL WELL-BEING</u>**	**Not at all**	**A little bit**	**Some-what**	**Quite a bit**	**Very much**
GF1	I am able to work (include work at home)	0	1	2	3	4
GF2	My work (include work at home) is fulfilling	0	1	2	3	4
GF3	I am able to enjoy life	0	1	2	3	4
GF4	I have accepted my illness	0	1	2	3	4
GF5	I am sleeping well	0	1	2	3	4
GF6	I am enjoying the things I usually do for fun	0	1	2	3	4
GF7	I am content with the quality of my life right now	0	1	2	3	4

Copyright 1987, 1997

FACIT-F (Version 4)

Please circle or mark one number per line to indicate your response as it applies to the past 7 days.

	ADDITIONAL CONCERNS	Not at all	A little bit	Some-what	Quite a bit	Very much
HI7	I feel fatigued	0	1	2	3	4
HI12	I feel weak all over	0	1	2	3	4
An1	I feel listless ("washed out")	0	1	2	3	4
An2	I feel tired	0	1	2	3	4
An3	I have trouble starting things because I am tired	0	1	2	3	4
An4	I have trouble finishing things because I am tired	0	1	2	3	4
An5	I have energy	0	1	2	3	4
An7	I am able to do my usual activities	0	1	2	3	4
An8	I need to sleep during the day	0	1	2	3	4
An12	I am too tired to eat	0	1	2	3	4
An14	I need help doing my usual activities	0	1	2	3	4
An15	I am frustrated by being too tired to do the things I want to do	0	1	2	3	4
An16	I have to limit my social activity because I am tired	0	1	2	3	4

Copyright 1987, 1997
