# Supplementary material for: Exploring the effects of a combined exercise programme on pain and fatigue outcomes in people with systemic sclerosis: study protocol for a large European multi-centre randomised controlled trial
Source: Trials. 2022 Nov 28;23:962. doi: 10.1186/s13063-022-06853-1 (PMC9706982; doi:10.1186/s13063-022-06853-1)
Supplement: Supplementary file 3 — Additional file 3. SscQoL Questionnaire [file 13063_2022_6853_MOESM3_ESM.pdf]

SSc-QoL

Date:

Name:____ Age: _______ Sex:_______

On the following pages you will find some statements which have been made by people who have suffered with Scleroderma

Instructions: This questionnaire consists of 29 statements. Please read each statement carefully, and then choose **True** if the statement applies to you and choose **Not True** if it does not apply to you **at the moment**. Circle the appropriate number.

1. **I can't do anything without really thinking it through**
   1 True
   0 Not True

2. **It's always on my mind**
   1 True
   0 Not True

3. **I worry that I let people down**
   1 True
   0 Not True

4. **My condition makes me angry**
   1 True
   0 Not True

5. **I would like to be spontaneous**
   1 True
   0 Not True

6. **I get upset when I can't do things**
   1 True
   0 Not True

7. **I often get frustrated**
   1 True
   0 Not True

8. **I cannot rely on how I will be tomorrow**
   1 True
   0 Not True

9. **My condition means I have disturbed sleep**
   1 True
   0 Not True

10. **It has affected me a lot socially**
    1 True
    0 Not True

11. **It has affected the health of people around me**
    1 True
    0 Not True

12. **My hands don't work as well as they did**
    1 True
    0 Not True

13. **It puts a strain on my personal relationships**
    1 True
    0 Not True

Continue on Back

©The University of Leeds 2012. SSc-QoL Version 2 (July 2012).

14. **I need to rest more often**

1 True

0 Not True

15. **I avoid certain social situations because I am embarrassed**

1 True

0 Not True

16. **I find it difficult to take care of the people I'm close to**

1 True

0 Not True

17. **I take to heart things which wouldn't have worried me before**

1 True

0 Not True

18. **Life is just not what it was**

1 True

0 Not True

19. **I can't cope at all**

1 True

0 Not True

20. **I feel very isolated**

1 True

0 Not True

21. **I am unable to join in activities with friends and family**

1 True

0 Not True

22. **Household tasks can be a problem**

1 True

0 Not True

23. **I have had to stop some of my hobbies**

1 True

0 Not True

24. **There are days when you are really tired and don't want to talk to anyone**

1 True

0 Not True

25. **I feel guilty at being ill**

1 True

0 Not True

26. **I struggle to wash myself as I would like**

1 True

0 Not True

27. **I feel helpless**

1 True

0 Not True

28. **Pain tires me out**

1 True

0 Not True

29. **I miss being able to sort things out**

1 True

0 Not True
